# Cognitive performance in relapsing remitting multiple sclerosis: A longitudinal study in daily practice using a brief computerized cognitive battery

**DOI:** 10.1186/1471-2377-11-68

**Published:** 2011-06-07

**Authors:** Chris Edgar, Peter J Jongen, Evert Sanders, Christian Sindic, Sophie Goffette, Michel Dupuis, Philippe Jacquerye, Daniel Guillaume, Regine Reznik, Keith Wesnes

**Affiliations:** 1 United BioSource Corporation, 9 Gatehampton Road, Goring-on-Thames, RG8 0EN, UK; 2 MS4 Research Institute, Ubbergseweg 34, 6522 KJ Nijmegen, the Netherlands; 3 Amphia Ziekenhuis, Molengracht 21, 4818 CK Breda, the Netherlands; 4 Cliniques Universitaires St. Luc, Université catholique de Louvain, Avenue Hippocrate 10, 1200 Brussels, Belgium; 5 Clinique Saint-Pierre, Avenue Reine Fabiola 9, 1340 Ottignies, Belgium; 6 Centre Neurologique et de Readaptation Fonctionnelle, 30 rue Champs des Alouettes, 4557 Fraiture-en-Condroz, Belgium

## Abstract

**Background:**

There is need for a cognitive test battery that can be easily used in clinical practice to detect or monitor cognitive performance in patients with multiple sclerosis (MS). In order to conduct, in this patient group, a preliminary investigation of the validity and utility of a brief computerized battery, the Cognitive Drug Research (CDR) battery, we longitudinally assessed cognition in patients with relapsing remitting (RR) MS.

**Methods:**

Forty-three mildly disabled, clinically active RRMS patients were repeatedly assessed with the Digit Symbol Substitution Test (DSST), Paced Auditory Serial Addition Test (PASAT) and five composite scores derived from the CDR computerized cognitive test system (CDR System): Power of Attention, Continuity of Attention, Quality of Working Memory, Quality of Episodic Memory and Speed of Memory. The Multiple Sclerosis Functional Composite (MSFC) and Expanded Disability Status Scale (EDSS) measured disability.

**Results:**

The composite scores from the CDR battery generally showed excellent test-retest reliability over the repeated assessments, though was low on occasions for the Quality of Working Memory and Quality of Episodic Memory measures. The CDR measures tended to be highly correlated with other measures of cognition (DSST and PASAT) and were also strongly related to disability (EDSS and MSFC). Baseline scores indicated large impairments to visual information processing speed and attention (DSST, Cohen's d 1.1; Power of Attention d 1.4 [reaction time on tasks of focussed and sustained attention]), and a moderate impairment both to sustained attention (Continuity of Attention d 0.6) and complex information processing speed (Speed of memory d 0.7 [reaction time on tasks of working and episodic Memory]), when compared to normative data derived from healthy volunteers enrolled in a series of separate, prior clinical trials. Working memory (Quality of Working Memory) and episodic memory (Quality of Episodic Memory) were unimpaired.

**Conclusions:**

Preliminary validation of the CDR System indicated that for most, but not all measures psychometric properties were adequate and the measures were related to disability (EDSS and MSFC) and other measures of cognition.

## Background

Cognitive disturbances are increasingly being recognized as a prominent feature of multiple sclerosis (MS) [1], occurring in about half of all patients [2] and in one third of patients with early relapsing remitting MS (RRMS) [3]. Impaired cognition is moderately associated with total lesion volumes [4], cortical lesions [5] and increase of cortical lesions over time on magnetic resonance imaging (MRI) [6]. More robust correlations have been found between cognitive function and whole brain atrophy [7,8] and regional gray matter atrophy [9]. The most frequently impaired domains are complex attention, information processing speed, memory and executive functions [3,10,11]. MS patients with problems in cognitive performance have increased odds of becoming unemployed [2]. Importantly, cognitive symptoms in early MS are predictive of disability several years later [12], and in benign RRMS failure on neuropsychological tests predicts clinical worsening over a 3-year period [13].

Two widely used and recommended neuropsychological test batteries have been developed for use in research and care of MS patients. The Brief Repeatable Neuropsychological Battery (BRNB) [14,15], which includes the Selective Reminding Test (SRT) [auditory/verbal memory], Controlled Oral Word Association (COWAT) [language], Paced Auditory Serial Addition Test (PASAT) [auditory processing speed, working memory], Symbol Digit Modalities Test (SDMT) [visual processing speed, working memory], and the 10/36 Spatial Recall Test [visuo-spatial memory]. This battery has alternate forms and has been validated in several cultures and languages. A second battery, the Minimal Assessment of Cognitive Functioning in Multiple Sclerosis (MACFIMS) [16], was the result of a consensus conference and is an expansion of the BRNB, replacing the 10/36 with the Brief Visuospatial Memory Test-Revised (BVMTR) and the SRT with the California Verbal Learning Test-Second Edition (CVLT2), which have more established psychometric properties, in particular with respect to alternate forms and test-retest reliability. In addition, the MACFIMS includes the Delis-Kaplan Executive Functioning System (D-KEFS) Sorting Test [executive function] and the Judgment of Line Orientation Test [spatial function].

Despite their status as well established batteries, further development in this area is warranted due to several factors, particularly in respect of the utility of BRNB and MACFIMS in clinical trials and patient care. Rater and patient burden are high, with the MACFIMS taking around 90 minutes to administer and both batteries containing a series of component tests and scores. These batteries require a high degree of expertise and standardized administration, and scoring may be difficult in large scale, multicentre, multi-national clinical trials. Perhaps of most importance, the batteries include no measurement of reaction time. In a disease where information processing speed during cognitive tasks is recognized as one of the primary deficits, the measures are not capable of separating information processing speed from other aspects of task performance. For example, impairment of motor function, information processing speed or working memory might affect Digit Symbol Substitution Test (DSST)/SDMT or PASAT scores; information processing speed or language or executive search impairment might affect COWAT scores; and information processing speed or memory impairment might affect SRT, CVLT2, 10/36 and BVMTR scores. However, these tasks cannot differentiate selective impairment of the different functions, which contribute to overall performance.

The Cognitive Drug Research (CDR) System is a brief, multiple repeatable, computerized battery of cognitive tests (http://www.unitedbiosource.com) [17,18]. Multiple alternate forms and availability in several languages make the battery suited to multi-national clinical trials use. The battery uses computer algorithms or rule sets to generate alternate forms of tests and randomizes these across repeated assessments, such that at each time-point in a study schedule each participant completes a different form of the test. The use of a simple two button response box minimizes the motor component of task performance and facilitates its use in populations with impaired motor control e.g. Parkinson's disease [17]. The CDR System is modular, including tests of attention and information processing speed (Simple reaction time, Choice reaction time and Digit vigilance tasks), verbal and visuo-spatial working memory (Numeric and Spatial working memory tasks) and verbal and visual episodic memory (Immediate and Delayed word recall [verbal responses are recorded by the administrator], Word recognition and Picture recognition tasks). Sensitivity indices (SI), ranging from zero (chance performance) to one (perfect accuracy), have been calculated for working memory and recognition tasks [19].

In this study we investigated the validity and utility of the CDR System by longitudinally assessing cognitive performance in RRMS patients with the established DSST and PASAT and comparing the results with those obtained by the CDR System.

## Methods

The study was performed in two general hospitals (Amphia Ziekenhuis, Breda, the Netherlands; Clinique St. Pierre, Ottignies, Belgium), one university hospital (Cliniques Universitaires St. Luc, Université catholique de Louvain, Brussels, Belgium) and two MS centres (MS Centre Nijmegen, Nijmegen, the Netherlands; Centre Neurologique et de Readaptation Fonctionnelle, Fraiture-en-Condroz, Belgium), and was ancillary to the FLAIR study, an investigator-initiated, international study on health-related quality of life (HR-QoL) and disability in RRMS patients during treatment with intramuscular (interferon-beta-1a INFβ-1a) (ClinicalTrials.gov identifier NCT00534261). Inclusion criteria were: (1) RRMS, (2) age 18-70 years, inclusive, (3) two relapses in the preceding 24 months, (4) disease duration at least 12 months, (5) EDSS 5.5 or less, (6) naive for INFβ, (7) written informed consent prior to any assessments not part of routine care. Exclusion criteria and details on study design and procedures have been reported [20]. The study was approved by the Independent Review Board, Amsterdam, the Netherlands and carried out in compliance with the Helsinki Declaration. The study was funded by Biogen Idec Netherlands.

### Assessments

Cognitive function was assessed using the DSST, the PASAT with 3 sec. interval (PASAT 3") [part of the Multiple Sclerosis Functional Composite (MSFC)] and the CDR computerized battery. The DSST is a widely used measure of visual information processing speed and working memory, complex visual scanning and sustained attention [21]. The PASAT 3" measures processing speed and working memory in the auditory/verbal sphere [22]. The CDR System is modular, and the selected battery measured attention and psychomotor/information processing speed (Simple reaction time, Choice reaction time and Digit vigilance tasks - both accuracy of responding and reaction time to visual stimulus presentation), verbal and visuo-spatial working memory (Numeric and Spatial working memory tasks) and verbal and visual episodic memory (Immediate and Delayed word recall, Word recognition and Picture recognition tasks) (see Additional file 1); and took around 15-20 minutes to complete. To minimize the motor requirement in responding, patient responses were recorded via a simple response box with two large buttons, one marked 'YES' and one marked 'NO', in the patient's own language. The patient was not required to use the computer keyboard or mouse and in the word recall tests, oral responses were recorded by the test administrator. Five composite domain scores were derived from the CDR battery: Power of Attention (a measure of attention and psychomotor/information processing speed summing reaction times from the Simple reaction time, Choice reaction time and Digit vigilance tasks), Continuity of Attention (a measure of attention summing accuracy and error measures from the Choice reaction time and Digit vigilance tasks), Quality of Working Memory (a measure of working memory summing accuracy measures from the Numeric and Spatial working memory tasks), Quality of Episodic Memory (a measure of episodic memory summing accuracy measures from the Immediate and Delayed word recall, Word recognition and Picture recognition tasks) and Speed of Memory (a measure of complex information processing speed summing reaction times from the Numeric and Spatial working memory and Word and Picture recognition tasks) [23] (see Additional file 2). The average of z-scores for all individual task measures yielded the CDR composite.

Disability was measured by the MSFC [average of z-scores for PASAT 3", Timed 25-Foot Walk (Timed-25FWT) and 9-Hole Peg Test (9-HPT)] [24] and the Expanded Disability Status Scale (EDSS) [25].

Physical and mental domains of HR-QoL were measured by the Multiple Sclerosis Quality of Life-54 (MSQoL-54) questionnaire. Scores for each domain range from 0 to 100, where higher values indicate better HR-QoL.

The CDR battery, the DSST and the MSFC were performed at Day -60 (training), Day -30 (training), Day 0 (baseline), Day 30 and Months 3, 6, 12, 18 and 24. MSQoL-54 scores were assessed at Day 0 and Months 3, 6, 12, 18 and 24, and the EDSS score on Day 0 and Month 24. Training was performed prior to the baseline assessment to familiarize patients with the procedures and overcome initial learning/practice effects.

### Statistical Analyses

Validity of the CDR battery was evaluated by assessing: a) test-retest reliability (Pearson correlation between subsequent assessments); b) practice effects (using the ANOVA analyses from the model described below); c) concurrent validity (Pearson correlation of cognitive measures with Physical MSQoL-54, Mental MSQoL-54 and EDSS scores at screening/baseline and Month 24; and Pearson correlation between CDR cognitive measures and DSST and MSFC measures at screening/baseline and Month 24); and d) discriminant validity (comparison to age-matched healthy controls, mean age 33.4 years, standard deviation [SD] 12.35, from the CDR normative database [version 3], derived from volunteers enrolled in a series of prior clinical trials). For the latter evaluation, the size of differences in outcome between patients and healthy controls was calculated using Cohen's d. Effect sizes may be interpreted as small (d ≥0.2), moderate (d ≥0.5) or large (d ≥0.8).

Correction for multiple comparisons was made using the Bonferroni method at p = 0.05 for each set of analyses conducted (p-value following correction indicated in the table legends).

An additional analysis was conducted to determine the number of patients impaired on the CDR cognitive measures. Patients were classified as impaired if they were ≥ 1 SD below normative data on three or more of the five composite domain scores derived from the battery (Power of Attention, Continuity of Attention, Quality of Working Memory, Quality of Episodic Memory and Speed of Memory). T-tests were used to evaluate level of disability on the EDSS in impaired versus unimpaired patients. The ANOVA assessing change over time was repeated fitting impairment at Day -30 as a fixed effect and the interaction term between time-point and impairment.

Finally, analyses were conducted to assess the change over time in cognitive parameters.Changes over time were assessed using one-way analyses of covariance using a mixed model (SAS^® ^PROC MIXED) with a fixed effect term for time-point and a random effect for patients. Comparisons between the time-points were made using the t-test from the LSmeans statement.

## Results

### Patient characteristics

Forty-three RRMS patients were studied, 30 female and 13 male. Mean age was 38.8 years (SD 10.5) and mean EDSS score 2.8 (SD 1.15). Mean disease duration was 6.0 years (SD 5.7), mean time since diagnosis 3.3 years (SD 4.1), and mean annualized relapse rate over the prior 24 months 1.2 (SD 0.4).

### Test-retest reliability

For most cognitive measures test-retest reliability was good (>0.7) and statistically significant. Exceptions were Quality of Working Memory and Quality of Episodic Memory, which showed lower and more variable correlation coefficients (Tables 1 and 2).

**Table 1:** Test-retest reliability between successive assessments

	Day -60 to -30	Day -30 to 0	Day 0 to 30	Day 30 to Month 3	Month 3 to 6	Month 6 to 12	Month 12 to 18	Month 18 to 24
Power of Attention	0.90 p < 0.0001	0.90 p < 0.0001	0.90 p < 0.0001	0.92 p < 0.0001	0.94 p < 0.0001	0.92 p < 0.0001	0.90 p < 0.0001	0.86 p < 0.0001

Continuity of Attention	0.76 p < 0.0001	0.87 p < 0.0001	0.72 p < 0.0001	0.9 p < 0.0001	0.87 p < 0.0001	0.88 p < 0.0001	0.91 p < 0.0001	0.89 p < 0.0001

Quality of working Memory	0.35 p = 0.0535	0.65 p < 0.0001	0.53 p = 0.0024	0.75 p < 0.0001	0.41 p < 0.0001	0.58 p = 0.0006	0.73 p < 0.0001	0.55 p = 0.0012

Quality of Episodic Memory	0.52 p = 0.0262	0.7 p = 0.0012	0.5 p = 0.0331	0.73 p = 0.0006	0.66 p = 0.0027	0.82 p < 0.0001	0.69 p = 0.0015	0.7 p < 0.0001

Speed of Memory	0.92 p < 0.0001	0.95 p < 0.0001	0.88 p < 0.0001	0.91 p < 0.0001	0.88 p < 0.0001	0.89 p < 0.0001	0.83 p < 0.0001	0.84 p < 0.0001

CDR composite	0.92 p < 0.0001	0.95 p < 0.0001	0.94 p < 0.0001	0.97 p < 0.0001	0.9 p < 0.0001	0.93 p < 0.0001	0.93 p < 0.0001	0.96 p < 0.0001

DSST	0.97 p < 0.0001	0.96 p < 0.0001	0.96 p < 0.0001	0.96 p < 0.0001	0.96 p < 0.0001	0.96 p < 0.0001	0.96 p < 0.0001	0.96 p < 0.0001

CDR, Cognitive Drug Research, DSST, Digit Symbol Substitution Test. Correcting for multiplicity p-values are statistically significant at p < 0.0006.

**Table 2:** Test-retest reliability between successive assessments

	Day -60 to -30	Day -30 to 0		Day 0 to Month 3	Month 3 to 6	Month 6 to 12	Month 12 to 18	Month 18 to 24
Timed 25-FWT	0.95 p < 0.0001	0.96 p < 0.0001		0.91 p < 0.0001	0.96 p < 0.0001	0.94 p < 0.0001	0.72 p < 0.0001	0.97 p < 0.0001

9-HPT	0.96 p < 0.0001	0.92 p < 0.0001		0.91 p < 0.0001	0.92 p < 0.0001	0.93 p < 0.0001	0.95 p < 0.0001	0.95 p < 0.0001

PASAT 3"	0.85 p < 0.0001	0.92 p < 0.0001		0.84 p < 0.0001	0.91 p < 0.0001	0.92 p < 0.0001	0.94 p < 0.0001	0.97 p < 0.0001

MSFC	0.96 p < 0.0001	0.96 p < 0.0001		0.93 p < 0.0001	0.96 p < 0.0001	0.95 p < 0.0001	0.9 p < 0.0001	0.98 p < 0.0001

Timed 25-FWT, Timed 25 Foot Walk Test; 9-HPT, 9 Hole Peg Test, PASAT 3", Paced Auditory Serial Addition Test 3 sec.; MSFC, Multiple Sclerosis Functional Composite. Correcting for multiplicity p-values are statistically significant at p < 0.0006.

### Correlations of cognitive and MSFC measures with EDSS and MSQoL-54

For EDSS at screening (Day -60) the largest correlation coefficient was seen for Power of Attention (0.62), followed by the CDR composite (0.59), and 9-HPT (0.55). For EDSS at Month 24 the largest correlation coefficient was seen for DSST (0.76), followed by the CDR composite (0.61), and MSFC (0.56). For MSQOL-54 Physical at screening the largest coefficient was seen for Timed 25-FWT (0.35) and all correlations were non-significant and small. For MSQOL-54 Physical at Month 24 the largest correlation coefficient was seen for Quality of Episodic Memory (0.64), followed by the Quality of Working Memory (0.35), and Continuity of Attention (0.34) and all correlations were non-significant. For MSQOL-54 Mental at screening the largest coefficient was seen for 9-HPT (0.23) and all correlations were non-significant and small. For MSQOL-54 Physical at Month 24 the largest correlation coefficient was seen for Quality of Episodic Memory (0.6), followed by the Quality of Working Memory (0.41), the CDR composite (0.25) and again all correlations were non-significant (Table 3).

**Table 3:** Correlations of cognitive and MSFC measures with physical and mental MSQOL-54 and EDSS at screening (Day 0) and Month 24

	MSQOL-54 Physical		MSQOL-54 Mental		EDSS	
	**Day 0**	**Month 24**	**Day 0**	**Month 24**	**Day -60**	**Month 24**

Power of Attention	0.14 p = 0.4371	0.21 p = 0.2707	0.1 p = 0.5311	0.07 p = 0.6964	0.62 p < 0.0001	0.5 p = 0.0023

Continuity of Attention	0.16 p = 0.2395	0.34 p = 0.0755	0.04 p = 0.8276	0.04 p = 0.8132	0.43 p = 0.0062	0.53 p = 0.001

Quality of working Memory	0.06 p = 0.733	0.35 p = 0.0598	0.14 p = 0.3742	0.41 p = 0.017	0.48 p = 0.0018	0.37 p = 0.0276

Quality of Episodic Memory	0.01 p = 0.9654	0.64 p = 0.0034	0.02 p = 0.9028	0.6 p = 0.0029	0.33 p = 0.0422	0.35 p = 0.0892

Speed of Memory	0.18 p = 0.3038	0.42 p = 0.0239	0.02 p = 0.8819	0.19 p = 0.2949	0.45 p = 0.0035	0.43 p = 0.0092

CDR composite	0.19 p = 0.2711	0.47 p = 0.0412	0.13 p = 0.4107	0.25 p = 0.2676	0.59 p < 0.0001	0.61 p = 0.0015

DSST	0.24 p = 0.1618	0.27 p = 0.2395	0.09 p = 0.5748	0.06 p = 0.7577	0.47 p = 0.0028	0.76 p < 0.0001

Timed 25-FWT	0.35 p = 0.0373	0.27 p = 0.154	0.15 p = 0.343	0.07 p = 0.6786	0.0 p = 0.9764	0.49 p = 0.002

9-HPT	0.0 p = 0.9896	0.08 p = 0.6629	0.23 p = 0.12	0.08 p = 0.652	0.55 p = 0.0001	0.53 p = 0.0003

PASAT 3"	0.03 p = 0.8805	0.16 p = 0.411	0.07 p = 0.6612	0.06 p = 0.7142	0.4 p = 0.0071	0.42 p = 0.0091

MSFC	0.17 p = 0.3352	0.19 p = 0.3328	0.02 p = 0.9241	0.12 p = 0.4834	0.43 p = 0.0044	0.56 p = 0.0003

MSFC, Multiple Sclerosis Functional Composite; MSQOL-54, Multiple Sclerosis-54 Quality of Life; EDSS, Expanded Disability Status Scale; CDR, Cognitive Drug Research, DSST, Digit Symbol Substitution Test; Timed 25-FWT, Timed 25 Foot Walk Test; 9-HPT, 9 Hole Peg Test, PASAT 3", Paced Auditory Serial Addition Test 3 sec. Correcting for multiplicity p-values are statistically significant at p < 0.0008.

### Correlations of CDR scores with DSST and MSFC measures

At baseline the CDR composite correlated well with the DSST and the PASAT 3", as well as with the 9-HPT and the MSFC, as did Power of attention (Table 4). Correlations with leg function (Timed 25-FWT) were less strong.

**Table 4:** Correlations between CDR measures and DSST, MSFC component scores and MSFC at baseline

	Power of Attention	Continuity of Attention	Quality of working memory	Quality of episodic memory	Speed of memory	CDR composite
DSST	0.61 p < 0.0001	0.49 p = 0.0009	0.33 p = 0.0333	0.25 p = 0.1068	0.55 p = 0.0001	0.7 p < 0.0001

Timed 25-FWT	0.18 p = 0.236	0.23 p = 0.1465	0.31 p = 0.0424	0.14 p = 0.3678	0.15 p = 0.3217	0.17 p = 0.2673

9-HPT	0.55 p = 0.0001	0.32 p = 0.0368	0.12 p = 0.3767	0.16 p = 0.3035	0.4 p = 0.0076	0.48 p = 0.001

PASAT 3"	0.6 p < 0.0001	0.31 p = 0.0431	0.2 p = 0.2092	0.17 p = 0.2692	0.35 p = 0.0209	0.49 p = 0.0009

MSFC	0.61 p = 0.0001	0.39 p = 0.0095	0.29 p = 0.0549	0.09 p = 0.5752	0.42 p = 0.0056	0.52 p = 0.0003

CDR, Cognitive Drug Research; DSST, Digit Symbol Substitution Test; MSFC, Multiple Sclerosis Functional Composite; Timed 25-FWT, Timed 25-Foot Walk Test; 9-HPT, 9 Hole Peg Test, PASAT 3", Paced Auditory Serial Addition Test 3 sec. Correcting for multiplicity p-values are statistically significant at p < 0.0017.

### Baseline cognitive function

CDR and DSST data at baseline were compared to normative data (CDR data base version 3) derived from healthy age matched volunteers, using data gathered in a series of prior clinical trials. Effect size differences (Cohen's d) showed large impairments to Power of Attention (d = 1.4) and DSST (d = 1.1), and moderate impairments to Continuity of Attention (0.6) and Speed of Memory (0.7). Quality of Working Memory and Quality of Episodic Memory were unimpaired (Table 5).

**Table 5:** Cognition at baseline versus normative data

	Mean (SD)		d	p
Measure	Normative Data (N≥1688)	RRMS (N = 43)		

Power of Attention	1083 (107)	1228 (162)	1.4	<0.0001

Continuity of Attention	64 (11.3)	57.3 (11.9)	0.6	0.0006

Quality of Working Memory	1.78 (0.21)	1.82 (0.16)	-0.2	0.1183

Quality of Episodic Memory	186 (53)	180 (56)	0.1	0.5296

Speed of Memory	3106 (698)	3589 (850)	0.7	0.0006

DSST^	58.7 (17.8)	41.5 (9.6)	1.1	<0.0001

SD, Standard Deviation; d, Cohen's d (effect size); RRMS, Relapsing Remitting Multiple Sclerosis; DSST, Digit Symbol Substitution Test. ^DSST Normative Data N = 151; < 0.3 small effect size; 0.3 to 0.7 moderate effect size; > 0.7 large effect size. Correcting for multiplicity p-values are statistically significant at p < 0.008.

### Level of cognitive impairment

The number of patients with cognitive impairment, defined as three or more domains ≥ 1 SD below age matched normative data, was 14 at Day -30 (33%) and 16 (41%) at Month 24. Learning/practice effects may miss-categorise a small number of patients if training/familiarisation is not conducted, with 17 patients (39.5%) categorized as impaired at the initial Day -60 time-points. As expected, the presence of cognitive impairment was associated with a statistically significantly greater disability on the EDSS (Table 5). Using cognitive impairment as a fixed effect in the ANOVA model we showed, as would be expected, a highly significant effect of impairment, with the cognitively impaired patients performing more poorly on all measures. However, no interaction was evident with time-point and there was no support for cognitive impairment at 'baseline' (Day -30) predicting subsequent decline in cognition in these patients (Table 6).

**Table 6:** Number of patients impaired by ≥ 1 SD on three or more cognitive domains from the CDR System

Patient Impaired in Three or More Cognitive Domains?	Day -60		Month 24	
	N (%)	EDSS mean (SD)	N (%)	EDSS mean (SD)

No	26 (60.5)	2.4 (1.15)	23 (59)	2.4 (1.46)

Yes	17 (39.5)	3.4 (0.84)	16 (41)	3.3 (1.27)

T-test on EDSS		t 3.34 [40] p = 0.0018		t 2.06 [35] p = 0.0469

SD, Standard Deviation. Correcting for multiplicity p-values are statistically significant at p < 0.025.

CDR System domain scores: Power of Attention; Continuity of Attention; Quality of Working Memory; Quality of Episodic Memory; and Speed of Memory.

### Change over time

**Figure 1:**
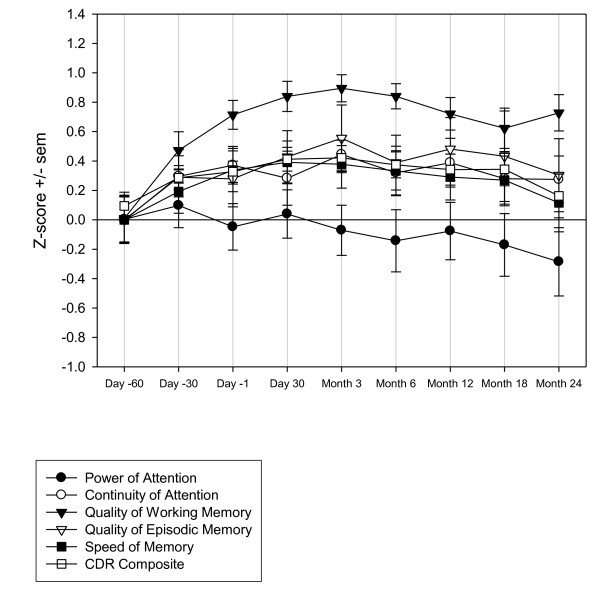
**Z-score change for CDR System cognitive measures (Z-score calculated as (X-mean)/SD at Day -60)**. CDR, Cognitive Drug Research; SD, standard deviation.

**Figure 2:**
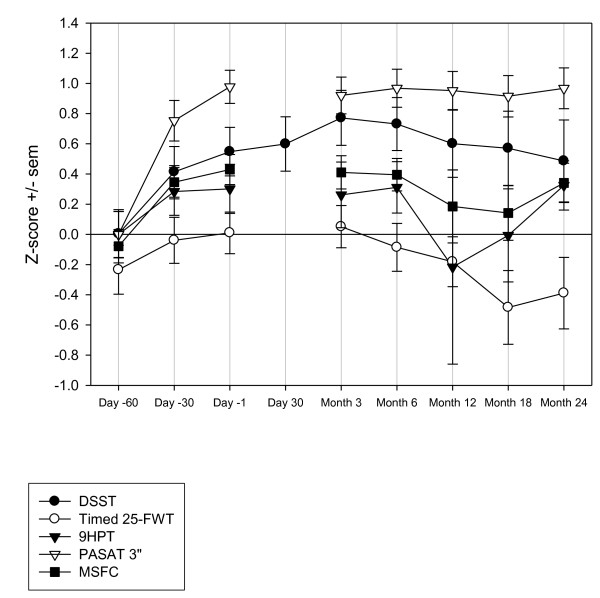
**Z-score change for DSST, MSFC Components and MSFC (Z-score calculated as (X-mean)/SD at Day -60)**. DSST, Digit Symbol Substitution Test; MSFC, Multiple Sclerosis Functional Composite; SD, standard deviation; Timed 25-FWT, Timed 25 Foot Walk Test; 9HPT, 9 Hole Peg Test, PASAT 3", Paced Auditory Serial Addition Test 3 sec.

For several measures improvements occurred during the screening phase, most notably for the PASAT 3" and Quality of Working Memory, which showed statistically significant main effects of time-point in the ANOVA model. For Quality of Working Memory the LSmeans comparisons between each time-point and the subsequent assessment showed a single statistically significant difference between Day -60 and Day -30 (t 2.85 [300] p = 0.0046). For PASAT 3" the LSmeans comparisons between each time-point and the subsequent assessment also showed a single statistically significant difference between Day -60 and Day -30 (t 4.17 [266] p < 0.0001). In general terms, changes were most marked between Days -60 and -30 and indicated learning/practice effects. The changes displayed a typical 'power-curve' with increasingly smaller improvements over the repeated assessments (Figures 1 and 2 and Table 7).

**Table 7:** Change over time for CDR domain scores, CDR composite, DSST, MSFC components and MSFC

	Main Effect of Time-point
Power of Attention	F 0.39 [8,300] p = 0.928

Continuity of Attention	F 0.68 [8,300] p = 0.708

Quality of Working Memory	F 5.6 [8,300] p < 0.0001

Quality of Episodic Memory	F 0.64 [8,300] p = 0.7458

Speed of Memory	F 0.67 [8,300] p = 0.7157

CDR composite	F 1.42 [8,274] p = 0.1876

DSST	F 1.48 [8,300] p = 0.1627

Timed 25-FWT	F 1.15 [7,267] p = 0.33

9-HPT	F 0.51 [7,265] p = 0.8238

PASAT 3"	F 6.96 [7,266] p < 0.0001

MSFC	F 1.68 [7,264] p = 0.1152

F, F ratio (PROC Mixed analysis of covariance output); CDR, Cognitive Drug Research, DSST, Digit Symbol Substitution Test; MSFC, Multiple Sclerosis Functional Composite; Timed 25-FWT, Timed 25 Foot Walk Test; 9-HPT, 9 Hole Peg Test, PASAT 3", Paced Auditory Serial Addition Test 3 sec. Correcting for multiplicity p-values are statistically significant at p < 0.005.

## Discussion

The CDR System has previously been validated in dementia [17] and traumatic brain injury [18], and is used in a variety of disease states and cognitive disorders including dementia, epilepsy and sleep disorders, to demonstrate both efficacy and safety of drugs [26-28]. The battery uses alternate forms of tests and randomizes these across repeated assessments. It is important to note that these alternate forms have not been specifically evaluated to demonstrate equivalence i.e. that they are parallel forms. However, the forms are as far as possible conceptually equivalent and the use of randomization prevents systematic bias in comparison between visits when comparing between or within groups. In the present study population, test-retest reliability was moderate to high for most CDR measures and the measures correlated with other assessments of cognition (DSST, PASAT 3") as well as with disability, supporting the validity of the battery in RRMS. With the exception of two measures test-retest ranged between 0.72 and 0.98 and thus could be considered high. The two measures showing more variable test-retest were Quality of working (0.35 to 0.75) and Quality of episodic memory (0.5 to 0.82). The poorest of these might possibly be related to the learning/practice effects on the Quality of working memory measure, as this was seen between the first and second assessments, where the largest improvement occurred. However, the possibility for non-equivalence of alternate forms to influence test-retest must also be considered. It was of note that the CDR measures were correlated with the more widely used PASAT. The PASAT, as a component of both the MSFC, BRNB and MACFIMS, has been extensively used to study cognition in MS and is thought primarily to measure information processing speed deficits [29]. The DSST, though not widely used in MS, is a common cognitive test in which the patient copies symbols paired with numbers against a time limit and is the reverse of the SDMT, in which the patient copies numbers paired with symbols. The SDMT using verbal responses is also included in the BRNB and MACFIMS batteries and may measure similar aspects of cognitive function to the PASAT and DSST, particularly information processing speed; and has been proposed as a replacement for the PASAT in the MSFC [30,31].

Importantly, ease of use and the automation of the CDR System facilitate cognitive assessment in a daily care setting, and electronic data capture and computer systems validation enhance data quality. In comparison to the BRNB and MACFIMS batteries, the selected CDR battery was shorter in duration, reducing patient burden, though it does not cover some aspects of function such as visual recall and abstract problem solving; and component measures do not need to be hand scored and entered into datasets, reducing rater burden and making the tests better suited to clinical trials or patient care. Recently, another computerized battery, the Automated Neuropsychological Assessment Metrics (ANAM), has been reported to be sensitive to cognitive impairment in MS patients [32]. In our study, the CDR battery identified impairment to information processing speed (d Power of Attention 1.4, d Speed of Memory 0.7) and attention (d Continuity of Attention 0.6) as compared to control data. The size of these impairments was highly consistent with prior findings in MS, in comparable cognitive domains. A study in MS patients (N = 65) versus controls (N = 46), identified impairment to visual processing speed and working memory (d SDMT 1.3) and auditory processing speed and working memory (d PASAT 0.7 to 0.9) [2]. However, it should be noted we compared the present data to normative data from healthy volunteers combined from a series of prior industry sponsored clinical trials, as opposed to a matched control sample gathered as part of the present study. As such although age matched, the samples will differ in respect of dimensions other than RRMS diagnosis that may be relevant to cognitive performance.

The CDR measures were correlated with disability (EDSS), in particular Power of Attention and the CDR composite, which showed a comparable relationship to the EDSS as the DSST and the MSFC, which incorporates both measures of cognitive function and arm and leg function i.e. disability. In addition, those patients characterized as cognitively impaired using the CDR battery had greater disability scores on the EDSS (Table 6). However, associations with HR-QoL were weaker. As expected, correlations were evident between the CDR battery measures and other measures assessing aspects of cognition (DSTT and PASAT 3"), but were not seen with leg function (Timed 25-FWT).

The lack of impairment to memory in our patient group was not consistent with prior findings indicating memory impairment to be prominent in MS. This could reflect properties of the CDR memory measures themselves e.g. sensitivity and/or lacking in sufficient difficulty. Alternatively, the study population might have been different from that in other studies e.g. relatively well educated with respect to the normative sample and thus 'cognitive reserve' might account for the lack of memory impairment. The CDR tasks of delayed and immediate recall and Spatial working memory nominally cover the same cognitive domains as tasks included in the BRNB and MACFIMS, which have identified impairments in MS patient populations. Thus, in conjunction with findings which show heterogeneity in cognitive impairment in MS [33], it is possible that the present sample may have presented an atypical pattern of impairment. Additionally, the CDR System has no measure of visual recall, as included in the MACFIMS and that may be particularly sensitive in this population. It would be important for future studies employing the CDR System to collect data on education, employment history and other potentially relevant demographics. However, it should be noted that some patients were at ceiling on accuracy measures for Spatial and Numeric working memory, Word and Picture recognition and PASAT 3". Thus these tasks may not be sufficiently challenging. A further issue which will need to be clarified in future studies is a more complete clinical characterization of the MS population to address other factors that may also impact upon cognition such as depression and fatigue. Thus a follow-up study in a larger sample with extensively described demographic and clinical variables is now necessary.

Practice effects are well known for the PASAT [34] and were also marked for the Quality of Working Memory from the CDR battery (Table 7). Our data confirm the importance of 'training' sessions for cognitive assessments prior to baseline [35,36], particularly in uncontrolled longitudinal studies, to overcome the large initial improvement in performance between the first and second administration of the tasks. Conclusions regarding any treatment effect cannot be drawn due to the fact that the assessments were conducted during an uncontrolled observational study. Thus without suitable control arms, it is not possible to differentiate potential treatment effects from those of the disease and/or properties of the measures themselves, over repeated assessments.

## Conclusions

The CDR System measures of attention, psychomotor/information processing speed, complex information processing speed and a global composite, showed good psychometric properties and were related to other measures of cognition and to disability. The data provide initial evidence for the utility and validity of the CDR System for use in MS clinical trials. To further validate the CDR System, data will be required in larger samples of patients with a more complete clinical and demographic characterization and with comparison to established cognitive/neuropsychological test batteries e.g. BRNB or MACFIMS.

## Competing interests

Dr. Edgar is an employee of United BioSource Corporation, owners of the CDR System cognitive battery, and has acted as a consultant to Astellas Pharma, Bristol-Myers Squibb, Debiopharm and Memory Pharm.

Dr. Jongen has received honoraria from Sanofi-Aventis, Teva, Merck-Serono, Novartis, Bayer-Schering, Biogen Idec and Allergan for activities as speaker, advisory committee member, research support, or travel grants for conferences.

Dr. Wesnes is also an employee of United BioSource Corporation, and has acted as a consultant to Astellas, Roche and Bristol Myers Squibb.

## Authors' contributions

CE contributed to conception and design of the study, performed data analysis, contributed to interpretation of data, drafted the manuscript, and has given final approval of the version to be published, PJJ initiated the study, contributed to conception and design of the study, acquisition of data, analysis of data and interpretation of data, drafted the manuscript, and has given final approval of the version to be published, ES contributed to conception and design of the study, acquisition of data, interpretation of data, revised the manuscript critically for important intellectual content, and has given final approval of the version to be published, CS contributed to conception and design of the study, acquisition of data, interpretation of data, revised the manuscript critically for important intellectual content, and has given final approval of the version to be published, SG contributed to acquisition of data, interpretation of data, revised the manuscript critically for important intellectual content, and has given final approval of the version to be published, MD contributed to acquisition of data, interpretation of data, revised the manuscript critically for important intellectual content, and has given final approval of the version to be published, PJ contributed to acquisition of data, interpretation of data, revised the manuscript critically for important intellectual content, and has given final approval of the version to be published, DG contributed to acquisition of data, interpretation of data, revised the manuscript critically for important intellectual content, and has given final approval of the version to be published, RR contributed to acquisition of data, interpretation of data, revised the manuscript critically for important intellectual content, and has given final approval of the version to be published, and KW contributed to conception and design of the study, contributed to interpretation of data, revised the manuscript critically for important intellectual content, and has given final approval of the version to be published.

## Authors' information

Chris Edgar is a Clinical Lead with United BioSource Corporation, owners of the CDR System, and works in the development and delivery of clinical products/services related to clinical trials, such as rater training, data surveillance, and cognitive testing. Dr. Edgar has over 10 years experience in clinical trials, primarily in the use of cognitive/behavioural assessments in central nervous system and neuroscience drug development. Prior to working with United BioSource Corporation, Dr. Edgar was an independent consultant working on protocol development, central review of rating scales and scientific writing projects for clinical trials in all phases of development. Dr. Edgar is an expert in computerized cognitive assessment and a former Scientific Director at CDR Ltd, developers of the CDR System computerized cognitive test battery. Dr. Edgar has a PhD in Cognitive Psychopharmacology from the University of Northumbria and a Masters in Research Methods from Reading University, in the U.K.

Peter J Jongen is a neurologist and founding director of the MS4 Research Institute, Nijmegen, the Netherlands. He has been involved in MS clinical research and patient care for more than 15 years. He is member of the International Medical and Scientific Board of the Multiple Sclerosis International Federation (MSIF), former director of the MS Centre Nijmegen, former council member of the European Committee for Treatment and Research in Multiple Sclerosis (ECTRIMS), and author of over 90 peer-reviewed scientific articles. The MS4 Research Institute conceives, performs and coordinates scientific research on the therapeutic value of treatments in MS.

## Pre-publication history

The pre-publication history for this paper can be accessed here:

http://www.biomedcentral.com/1471-2377/11/68/prepub

## Supplementary Material

Additional file 1 describes the CDR System tasks and outcome measures Click here for file

Additional file 2 describes the derivation of cognitive domain scores Click here for file
