# Supplementary material for: Cognitive performance in relapsing remitting multiple sclerosis: A longitudinal study in daily practice using a brief computerized cognitive battery
Source: BMC Neurol. 2011 Jun 7;11:68. doi: 10.1186/1471-2377-11-68 (PMC3128855; doi:10.1186/1471-2377-11-68)
Supplement: Additional file 1 — describes the CDR System tasks and outcome measures [file 1471-2377-11-68-S1.DOC]

**Additional files**

**Additional file 1 - Cognitive Drug Research (CDR) System Tasks and Outcome Measures**

| Task Title | Task Description | Outcome Measures |
| --- | --- | --- |
| Practice Choice Reaction Time | The volunteer is required to respond to the words 'YES' and 'NO' as they appear on screen by pressing the corresponding button as quickly as possible. There are 20 trials during which each stimulus word is chosen randomly with equal probability; there is a varying inter-stimulus interval of between 1 and 3.5 seconds. The task lasts approximately 1 minute. | Choice Reaction Time Accuracy (%) [percentage of correct responses] |
|  |  | Choice Reaction Time (ms) [mean reaction time of correct responses] |
|  |  |  |
|  |  |  |
|  |  |  |
| Word Presentation | A list of 15 words is presented on the screen at the rate of 1 every 2 seconds for the volunteer to remember. The duration of the presentation is 30 seconds and no data are recorded. | N/A |
| Immediate Word Recall | Immediately after the last word is presented, the volunteer is given one minute to recall as many of the words as possible. | Immediate Word Recall Accuracy (%) [percentage of correct responses] |
|  |  | Immediate Word Recall Words Correctly Recalled (#) [number of correct responses] |
|  |  | Immediate Word Recall Errors (#) [number of errors of commission] |
| Picture Presentation | A series of 20 pictures, selected on a pseudo-random basis from 20 categories, including scenes and objects, is presented on the screen at the rate of 1 picture every 3 seconds, for the volunteer to remember. The duration of the presentation is 1 minute and no data are recorded. | N/A |
| Simple Reaction Time | The volunteer is instructed to press the 'YES' response button, as quickly as possible, every time the word 'YES' was presented on the screen. Fifty stimuli are presented with a varying inter-stimulus interval of between 1 and 3.5 seconds. The task lasts approximately 1.5 minutes. | Simple Reaction Time (ms) [mean reaction time of responses] |
|  |  |  |
|  |  |  |
|  |  |  |
| Digit Vigilance | A target digit is pseudo-randomly selected and constantly displayed to the right of the screen. A series of 450 digits is then presented in the centre of the screen at the rate of 150 per minute. The volunteer is required to press the 'YES' button, as quickly as possible, every time a digit in the series matches the target digit. The task lasts for 3 minutes. | Digit Vigilance Targets Detected (%) [percentage of correct responses] |
|  |  | Digit Vigilance Speed (ms) [mean reaction time of correct responses] |
|  |  | Digit Vigilance False Alarms (#) [number of errors] |
|  |  |  |
| Choice Reaction Time | The volunteer is required to respond to the words 'YES' and 'NO' as they appear on screen by pressing the corresponding button as quickly as possible. There are 50 trials during which each stimulus word is chosen randomly with equal probability; there is a varying inter-stimulus interval of between 1 and 3.5 seconds. The task lasts approximately 1.5 minutes. | Choice Reaction Time Accuracy (%) [percentage of correct responses] |
|  |  | Choice Reaction Time (ms) [mean reaction time of correct responses] |
|  |  |  |
|  |  |  |
|  |  |  |
| Spatial Working Memory | A picture of a house is presented on the screen with 4 of the 9 windows lit, for 10 seconds. The volunteer has to memorize the position of the lit windows. For each of the 36 subsequent presentations of the house, the volunteer is required to decide whether or not the single window that was lit, had been lit in the original presentation. The volunteer responds by pressing the corresponding 'YES' or 'NO' button, as appropriate, as quickly as possible. The task lasts approximately 1 minute. | Spatial Working Memory Original Stimuli Accuracy (%) [percentage of correct responses to target stimuli] |
|  |  | Spatial Working Memory New Stimuli Accuracy (%) [percentage of correct responses to novel stimuli] |
|  |  | Spatial Working Memory Sensitivity Index (SI) [sensitivity index of correct responses] |
|  |  | Spatial Working Memory Speed (ms) [mean reaction time of correct responses] |
|  |  |  |
|  |  |  |
|  |  |  |
|  |  |  |
|  |  |  |
|  |  |  |
|  |  |  |
| Numeric Working Memory | A series of 5 digits is presented, one every 1.15 seconds, for the volunteer to hold in memory. This is followed by a series of 30 probe digits, for each of which the volunteer has to decide whether it has appeared in the original series and press the corresponding 'YES' or 'NO' response button as quickly as possible. The task lasts approximately 1 minute. | Numeric Working Memory Original Stimuli Accuracy (%) [percentage of correct responses to target stimuli] |
|  |  | Numeric Working Memory New Stimuli Accuracy (%) [percentage of correct responses to novel stimuli] |
|  |  | Numeric Working Memory Sensitivity Index (SI) [sensitivity index of correct responses] |
|  |  | Numeric Working Memory Speed (ms) [mean reaction time of correct responses] |
|  |  |  |
|  |  |  |
|  |  |  |
|  |  |  |
|  |  |  |
| Delayed Word Recall | The volunteer is again given one minute to recall as many of the words originally presented as possible. | Delayed Word Recall Accuracy (%) [percentage of correct responses] |
|  |  | Delayed Word Recall Words Correctly Recalled (#) [number of correct responses] |
|  |  | Delayed Word Recall Errors (#) [number of errors of commission] |
| Word Recognition | The original words from Word Presentation plus 15 distracter words are presented, one at a time, in a randomized order. For each word, the volunteer is required to indicate whether he/she recognizes it from the original list of words by pressing the corresponding 'YES' or 'NO' button as quickly as possible. Following the response, there is a delay of 1 second before the next word is presented. The task lasts approximately 1 minute. | Word Recognition Original Stimuli Accuracy (%) [percentage of correct responses to target stimuli] |
|  |  | Word Recognition New Stimuli Accuracy (%) [percentage of correct responses to novel stimuli] |
|  |  | Word Recognition Sensitivity Index (SI) [sensitivity index of correct responses] |
|  |  | Word Recognition Speed (ms) [mean reaction time of correct responses] |
|  |  |  |
|  |  |  |
|  |  |  |
|  |  |  |
|  |  |  |
|  |  |  |
|  |  |  |
| Picture Recognition | The original pictures from Picture Presentation plus 20 distracter pictures are presented, one at a time. For each picture, the volunteer is required to indicate whether he/she recognizes it from the original series by pressing the corresponding 'YES' or 'NO' button as quickly as possible. Following the response, there is a delay of 1 second before the next picture is presented. The task lasts approximately 1 minute. | Picture Recognition Original Stimuli Accuracy (%) [percentage of correct responses to target stimuli] |
|  |  | Picture Recognition New Stimuli Accuracy (%) [percentage of correct responses to novel stimuli] |
|  |  | Picture Recognition Sensitivity Index (SI) [sensitivity index of correct responses] |
|  |  | Picture Recognition Speed (ms) [mean reaction time of correct responses] |
|  |  |  |
|  |  |  |
|  |  |  |
|  |  |  |
|  |  |  |
|  |  |  |
|  |  |  |

The sensitivity index (SI) is calculated from the formula presented by Frey and Colliver (1973) and combines the accuracy scores for the original (target) as well as novel (distractor) stimuli.

Frey PW, Colliver JA (1973). Sensitivity and responsivity measures for discrimination learning. Learning and Motivation 4: 327-342.
