# Supplementary material for: Cognitive performance in relapsing remitting multiple sclerosis: A longitudinal study in daily practice using a brief computerized cognitive battery
Source: BMC Neurol. 2011 Jun 7;11:68. doi: 10.1186/1471-2377-11-68 (PMC3128855; doi:10.1186/1471-2377-11-68)
Supplement: Additional file 2 — describes the derivation of cognitive domain scores [file 1471-2377-11-68-S2.DOC]

**Additional file 2 - Derivation of Cognitive Domain Scores**

| Cognitive Domain Score | Derivation |
| --- | --- |
| *Power of Attention (ms)*  The sum of speed / reaction time scores from the attention tasks.  A measure of attention and psycho-motor/information processing speed. | Simple Reaction Time + Digit Vigilance Speed + Choice Reaction Time |
| *Continuity of Attention (#)*  The sum of accuracy and error scores from the attention tasks.  A measure of attention. | (Digit Vigilance Targets Detected *0.45) + (Choice Reaction Time Accuracy *0.5) – Digit Vigilance False Alarms |
| *Quality of Working Memory (SI)*  The sum of accuracy scores from the working memory tasks.  A measure of verbal and visuo-spatial working memory. | Spatial Working Memory Sensitivity Index + Numeric Working Memory Sensitivity Index |
| *Quality of Episodic Memory (#)*  The sum of accuracy scores from the episodic memory tasks.  A measure of verbal and visual learning and memory. | (Word Recognition Original Stimuli Accuracy + Word Recognition Novel Stimuli Accuracy - 100) + (Picture Recognition Original Stimuli Accuracy + Picture Recognition Novel Stimuli Accuracy - 100) + ((Immediate Word Recall Words Correctly Recalled - Immediate Word Recall Errors) * 100 / 15) + (( Delayed Word Recall Words Correctly Recalled - Delayed Word Recall Errors) * 100 / 15) |
| *Speed of Memory (ms)*  The sum of speed / reaction time scores from the working and episodic memory tasks.  A measure of information processing speed. | Spatial Working Memory Speed + Numeric Working Memory Speed + Word Recognition Speed + Picture Recognition Speed |
